# 15 years of transcriptomic analysis on endometrial receptivity: what have we learnt?

**DOI:** 10.1186/s40738-019-0059-7

**Published:** 2019-08-05

**Authors:** Soumaya Messaoudi, Imane EL Kasmi, Amelie Bourdiec, Kimberley Crespo, Laurence Bissonnette, Cecile Le Saint, François Bissonnette, Isaac-Jacques Kadoch

**Affiliations:** 1 Ovo r&d, Montreal, Quebec Canada; 2 Department of Obstetrics and Gynecology, University of Montreal Hospital Centre, Montreal, Quebec Canada

## Introduction

Over the course of the last four decades, IVF has allowed an increasing number of infertile couples the chance to conceive. Considering the extensive research and advances in ART, too many IVF attempts still do not result in a successful pregnancy [1, 2]. Embryo implantation is a crucial event in the establishment of a pregnancy. It is now clear that embryo implantation relies upon cross-talk and synchronicity between the implanting embryo and a receptive endometrium [3]. This embryo-maternal cross-talk involves an elaborate and coordinated network of communication via timely released embryonic, maternal-derived signals, and well-targeted actions [4]. If the clinical and culture conditions to obtain a “good quality” embryo are well advanced today, ER remains the last barrier in ART. When a high-quality embryo is transferred, impaired uterine receptivity is believed to be one of the major reasons behind failure of the establishment of pregnancy [5, 6].

It has been suggested in a few studies that up to two-thirds of implantation failures are due to defects in ER whereas the quality of the embryo itself is responsible for only one-third of failures [7, 8]. An endometrium is receptive to an embryo in a spatially and temporally restricted period called the window of implantation (WOI). In natural cycles, this period, occurring during the mid-secretory phase, is limited to approximately 48 h, starting around the seventh day after the LH (luteinizing hormone) surge [9, 10]. The dynamic transition from a non-receptive to a receptive endometrium is still poorly understood. Several reports have shown that ER is defined by specific changes in factors involved in adhesion, invasion, survival, growth, differentiation, decidualization and immuno-modulation. The correct spatio-temporal synthesis and balance of these various factors are thought to play an important role in human uterine preparation for implantation [4, 11]. Extensive efforts have been made to understand and characterize a receptive endometrium, from the first histological dating methods to the ‘omics’ technologies [3, 7, 12]. Several endometrial dating criteria have been commonly used in clinical practice. One or more panels of biomarkers, predictive of optimal ER, have been analyzed in blood and uterine fluid. Leukaemia inhibitor factor (LIF) is an example of a potential biomarker of the WOI [13]. However, this approach has been judged unsatisfactory by several studies because LIF measurements in serum do not reflect fertility status and similar conclusions were reported for other biomarkers [3, 14]. Cervical mucus has also been used to date ER by analyzing cytokines and growth factors produced by a receptive endometrium and their transport to the cervical mucus [15]. However, other studies have been unable to detect these growth factors in cervical secretions throughout the menstrual cycle and in these studies no correlation was observed between cytokine levels in cervico-vaginal secretions and serum and between the cytokines gene expression level in the secretory endometrium and the concentrations in serum [3, 16]. Another group evaluated ER during IVF cycles using three-dimensional power Doppler ultrasound [17]. However, Sterzik et al. concluded that ultrasonography is an inadequate method to predict ER in IVF cycles since neither the endometrial thickness nor the echogenic pattern correlate with histological findings [18].

As demonstrated, endometrial dating criteria have been questioned in various randomized studies. This has encouraged further investigation and application of new technologies to try to objectively diagnose ER. Omics technologies, such as transcriptomics, have been used to identify biomarkers of human endometrium [7, 19]. Based on the transcriptomic signature identified in these studies, only two ER diagnostic tools have been commercialized in order to personalize the frozen embryo transfer (FET): the ERA test (Endometrial Receptivity Array) [20] and the Win-Test (Window Implantation Test) [21].

In this review, we screened publications of the transcriptomic profiles of fertile and infertile women during the secretory phase of natural and stimulated cycles in order to understand lessons learned from endometrial gene profiling.

## Transcriptomics of endometrial receptivity

With the development of microarray technologies, global approaches have been pursued to identify novel genes and pathways involved in the acquisition of a receptive endometrium [3, 11]. To the best of our knowledge, since 2002, 23 studies have been published, reporting the transcriptome of the human endometrium during the WOI [19, 20, 22–37]. The studies reporting gene expression analysis to identify molecular biomarkers of ER either in natural or controlled ovarian stimulation cycles (COS) for fertile and infertile women are listed in Tables 1 and 2.

**Table 1:** Characteristics of the analyzed studies on endometrial transcriptomics for fertile women in natural and stimulated cycle

First author and reference	Participants population	N of patients	Age	Comparative	Region	Array	First sample (day, n)	Second sample (day, n)	N of genes up-regulated	N of genes down regulated
Fertile women										
Natural cycles										
Carson et al. [22]	Fertile volunteers	6	Not specified	ES vs MS	North America	HG U95A (Affymetrix)	LH + 2/4 (*n* = 3)	LH + 7/9 (*n* = 3)	323	370
Riesewijk et al. [28]	Fertile volunteers	10	23-39	ES vs MS	Europe	HG U95A (Affymetrix)	LH + 2 (*n* = 5)	LH + 7 (*n* = 5)	153	58
Mirkin et al. [26]	Fertile Oocyte donor	8	24–32	ES vs MS	North America	HG U95A v2 (Affymetrix)	LH +3 (*n* = 3)	LH +8 (*n* = 5)	49	58
Talbi et al. [37]	Normo-Ovulatory Women	28	23-50	ES vs MS	North America	HGU133 plus 2.0 (Affymetrix)	ES *n* = 3	MS *n* = 8	1415	1463
Haouzi et al. [1]	normoresponder patients	31	22-36	ES vs MS	Europe	HGU133 plus 2.0 (Affymetrix)	LH+2 *n* = 31	LH+7 *n* = 31	746	51
Allegra et al. [38]	patients who became pregnant	15	<35	Pregnant vs non- pregnant	Europe	Low Density Array technology	LH+7/9 *n* = 15		23	11
Van Vaerenbergh [40]	patient who became pregnant	1	24	MS vs pregnant	Europe	HGU133 plus 2.0 (Affymetrix)	LH+5/7 *n* = 1		4	-
Diaz-Gimeno et al. [20]	Fertile donors	88	22-39	ES vs MS	Europe	Agilent Whole Human Genome Oligo Microarray	LH+1/5 (*n* = 13)	LH+7 (*n* = 5)	143	95
Horcajadas et al. [24]	Fertile Oocyte donor	10	23-39	NC vs COH	Europe	HG_U133A (Affymetrix)	LH+2 (*n* = 5)	LH+7 (*n* = 14)	874	505
Stimulated cycles										
Mirkin et al. [27]	Fertile Oocyte donor	15	24-32	Ag vs Atg vs NC	North America	HG U95A v2 (Affymetrix)	LH+8 (*n* = 5)	hCG+9 (*n* = 10) (Ag=3, Atg=7)	24	7
Horcajadas et al. [24]	Fertile Oocyte donor	10	23-39	NC vs COH	Europe	HG_U133A (Affymetrix)	LH+7 (*n* = 7)	hCG+7 (*n* = 5)	281	277
Simon et al. [29]	Fertile Oocyte donor	31	18–35	Ag vs Atg vs NC	Europe	HG_U133A (Affymetrix )	LH+7 (*n* = 12)	hCG+2/7 (*n* = 14)	132	193
Horcajadas et al. [49]	healthy fertile cycling donors	49	23–39	NC vs CCOS	Europe	HG_U133A (Affymetrix )	LH+7 (*n* = 5)	hCG+7 (*n* = 5)	69	73
Koler et al. [41]	Fertile Oocyte donor	8	31-38	NC vs CCOS	Europe + North America	HG_U133A (Affymetrix )	LH+5	hCG+2	142	98
Haouzi et al. [23]	Normo-Ovulatory Women	21	30.9+ 3.3	NS vs CCOS	Europe	HGU133 plus 2.0 (Affymetrix)	hCG+2 (*n* = 21)	hCG+5 (*n* = 21)	777	221
Haouzi et al. [30]	normal-responder patients	21	-	Ag vs Atg vs NC	Europe	HGU133 plus 2.0 (Affymetrix	LH+2 (*n* = 21)	LH+2 (*n* = 21)		
							Ag hCG+2 (*n* = 7)	Ag hCG+5 (*n* = 7)	731	451
							Atg hCG+2 (*n* = 14)	Atg hCG+5 (*n* = 14)	634	210

**Table 2:** Characteristics of the analyzed studies on endometrial transcriptomics for infertile women in natural and stimulated Cycle

First author and reference	Participants population	N of patients	Age	Comparative	Region	Array	First sample (day, n)	Second sample (day, n)	N of genes up-regulated	N of genes down regulated
Infertile women										
Natural cycles										
Koler et al. [41]	women with unexplained infertility + fertile women	32	19-40	Infertile vs fertile	Europe	Array-Ready Oligo SetTM for the Human Genome Version 3.0 (Operon)	cd21 Infertile women (*n* = 4) fertile women (*n* = 12		25	288
Altmae et al. [31]	women with unexplained infertility	Infertile women (*n* = 4) Fertile women (*n* = 4)	30.5+4.0 31.8+3.8	Infertile vs fertile	Europe	Whole Human Genome Oligo Microarray (Agilent Technologies)	LH +7 Infertile women (*n* = 4) fertile women (*n* = 5)		145	115
Koot et al. [34]	women experiencing RIF	115	26-39	Infertile vs fertile	Europe	Human whole genome gene expression microarrays V2 (Agilent)	mid-luteal phase *n* = 115	303 genes predictive of RIF	303 genes predictive of RIF	
Stimulated cycles										
Liu et al. [25]	Infertile women with normal menstrual cycles	47	26 -38	NC vs CCOS	Hong Kong	HG_U133A (Affymetrix)	LH+7 *n* = 5	hCG+7 *n* = 8	244	159
Ruiz-Alonso et al. [36]	Patients with repeated implantation failure	110	23-51	RIF vs controls pWOI/Pwoi delayed/pWOI advanced	Europe	homemade ERA	P+5 LH+7		-	-
Altmae et al. [19]	Patients with repeated implantation failure	15	30.2 ± 4.3	Infertile vs fertile	Europe	Whole Human Genome Oligo Microarray (Agilent Technologies	LH+7 (*n* = 5) P+6 (*n* = 5)		443	446
Haouzi et al. [33]	Oocyte-donation recipient patients RIF patients	39	31-50	NC vs HRT hormone replacement therapy	Europe	HGU133 plus 2.0 (Affymetrix)	LH+7 (*n* = 7) P+5 (*n* = 7)		1814	477

*Abbreviations: EP* Early-proliferative, *ES* Early-Secretory, *Ag* Agonist, *Atg* Antagonist, *LH+* LH surge + days, *NC* Natural cycle, *COH* Controlled ovarian hyperstimulation, *MS* mid-secretory, *CCOS* Controlled ovarian stimulation, *PP* proliferative phase, *P+* Progesterone+ days, *HRT* hormone replacement therapy**,** *pWO* personalised window of implantation

The majority of these studies analyzed endometrial transcriptomic profiles by comparing gene-expression profile shift between the early- and the mid-secretory phase under natural cycles to identify biomarkers believed to be involved in ER acquisition [38]. Transcriptomic approaches have then been used to compare the receptive gene-expression profile between natural and stimulated cycles in order to understand the effect of ovarian stimulation on the ER.

The fertile women included in the studies were usually characterized by a regular cycle, a normal uterus, no endocrine abnormalities or abnormal early follicular gonadotrophin values, no endometriosis (revealed by laparoscopy or pathology reports), no inflammation within the endometrium in any of the specimens and a correlation between histological dating and LH timing. Patients were egg donors or referred for male infertility [20, 22, 23, 28, 37, 39–41]. The infertile women in the studies on the other hand, had a history of repeated implantation failure or unexplained infertility [25, 31, 34, 36, 42].

## Transcriptomics of endometrial receptivity of fertile women

### Natural cycles

Several studies have compared the gene-expression profile of human ER from a pre-receptive (LH + 1/5) to a receptive (LH + 7/9) stage in fertile patients within a natural cycle [20–22, 26, 28, 37].

All these studies showed a specifically modulated gene expression profile during the WOI. However, as shown in Table 1, the number of genes differentially expressed between the pre-receptive and the receptive endometrial phases is extremely variable across these studies, from 107 to 2878 genes. Talbi et al. reported a transcriptomic signature of 2878 genes: including 1415 up- and 1463 down-regulated genes [37]. Two other studies showed the same proportion of up- and down-regulated genes in the receptive endometrium: 323 up- and 370 down-egulated genes [22] and 49 up- and 58 down-regulated genes [26]. Haouzi et al. identified an exclusive transcriptomic signature, when they intersected their gene list, it significantly modulated with those from four other transcriptomic studies [22, 26, 28, 37], with 746 up- and [5] down-regulated genes during the WOI [21]. This signature profile has similar proportion to what Riesewijk et al. found with 153 up- and 58 down-regulated genes [28]. Only these two studies compared gene-expression profiles in the same patient in early and the mid-secretory phase, this is a very important consideration, since it minimizes the impact of interpatient variability [21, 28]. On the other hand, a study by Díaz-Gimeno et al. identified 143 up- and 95 down-regulated genes [20].Thus, an important transcriptional activation during the WOI was observed in only three studies [20, 21, 28].

Among all these studies only two genes were common, osteopontin, also called SPP1 (secreted phosphoprotein 1), a glycoprotein involved in cellular adhesion and migration during embryo implantation, and interleukin (IL) IL15, a progesterone-regulated gene expressed in endometrial stromal cells. This cytokine seems to be involved in stages immediately before, during and after the apposition step. In addition, it permits normal proliferation of the stroma [7].

In 2009, from their transcriptomic signature of 1012 genes, Haouzi et al. [21] used qRT-PCR to validate the expression levels of five genes specifically modulated during the WOI in natural cycles: four of which were reported for the first time as new biomarkers of ER. All five endometrial genes were over-expressed during the mid-secretory phase (LH + 7) compared to the early-secretory phase (LH + 2). These genes were laminin β3 (LAMB3), microfibrillar associated protein5 (MFAP5), angiopoietin-like 1 (ANGPTL1), prokineticin 1 (PROK1), and nuclear localized factor 2 (NLF2). They are functionally involved in the extracellular-matrix remodeling of the endothelial-cell microenvironment, angiogenesis and the formation of the endothelial fenestration. In order to develop a diagnostic tool for human ER assessment, the authors selected the 11 most up-regulated genes predictive of a receptive endometrial status [33, 43, 44], that were not listed in other transcriptomic studies [22, 26–28]. This diagnostic ER tool, called the Win-Test was the first to be commercialized [43]. Two years later, Díaz-Gimeno et al.*,* identified 238 genes shown to be differentially expressed in the transition from early- to the mid-secretory phase. They defined their ER transcriptomic signature by using 134 genes included in the customized microarray (ERA), a genomic diagnostic tool for endometrial dating and detecting endometrial origin pathologies [20]. This diagnostic tool has been modified several times over the years and recently became known as the NGS ERA Predictor 2.0 [45].

However, a comparison of ERA signature genes with a microarray designed with 126 genes for the diagnosis of endometrial disorders [46] showed that 61 genes are commonly shared by both designs. This 126 genes list also contains two genes that have been previously reported by others [21, 28, 37], namely LAMB3 and MFAP5. In addition, the ERA test shares 47 genes in common with the identified meta-signature of 57 genes as probable ER biomarkers, identified by in silico analysis from previously published expression profiling studies [19]. The functional analysis by gene ontology of the ERA signature reveals that their gene selections are involved in immune response, cytoskeleton proteins, cell adhesion, signal transduction, mitotic cycle and cellular proliferation. Another study on fertile women who became pregnant in a subsequent IVF cycle, analyzed the endometrial gene-expression profile at LH + 7/9, one or two menstrual cycles before their IVF cycle [39]. Only 6 genes: vascular endothelial growth factor A, phospholipase A2 group IIA, alkaline phosphatase, leukaemia inhibitory factor, nicotinamide N-methyltransferase, and stanniocalcin 1 showed a significant uniform expression among patients who subsequently became pregnant. For all the other genes analyzed, there were considerable differences in their expression levels amongst women who become pregnant. Van Vaerenbergh et al. [41] in a unique in vivo study, compared the gene expression profile of conceptional to non-conceptional mid-luteal endometrium. A total of 394 differentially expressed genes with 310 probe sets up- and 84 probe sets down-regulated were found. The major networks represented by these genes included post-translational modification, cell signaling, cell movement, cell development and haematologic system functioning.

In all these comparisons of the secretory phase of fertile women in natural cycles, gene-expression profiles are different. Disparities of the results may be explained by several factors as described in Table 1 such as: size of patient samples (between 6 and 88 samples), age of the patients (between 22 and 39 years old), location of the patients (Europe, North America), sampling time during the cycle, type of DNA microarrays containing different genomic information (30 000 genes for the Hu133P oligonucleotide microarrays versus 12 000 genes for the Hu95A), statistical methodologies, bioinformatic tools, samples compared within the same or different patients. Among all these studies, only two compared and analyzed gene-expression profiles in the same patient.

Despite the differences among all the studies performed, they all agreed on the existence of a specific transcriptomic profile during the WOI. These characteristic profiles suggest that a unique transcriptional process occurs to achieve a receptive endometrial phenotype [21].

### Stimulated cycles

The effect of stimulated cycles on ER transcriptomics have been investigated in order to understand the changes in the profiles of receptive endometrium during COS and to identify the ovarian stimulation protocol that minimizes the impact on ER [20].

The majority of these studies have compared stimulated cycles (agonist or antagonist) with natural cycles in fertile women [23, 24, 40], whereas others have compared the effect between agonist and antagonist protocols [27, 29, 30] notable differences are observed when analyzing during the WOI.

Reports comparing stimulated with natural cycles demonstrated that the endometrium in stimulated cycles does not reach the state of receptivity in the same way as in natural cycles [23, 24, 29, 40, 47]. In 2008, Horcajadas et al. reported that COS induced a functional genomics delay of the receptive endometrium in comparison with natural cycles [40]. The authors compared the endometrial profiles of stimulated and natural cycles during the transition from the pre-receptive (LH/human chorionic gonadotrophin (hCG) + 1 until LH/hCG + 5) to the receptive phase (LH/hCG + 7 and LH/hCG + 9), and noted that the hCG + 9 profile relates more closely to the LH + 7 than the hCG + 7 profile. However, in the pre-receptive phase the endometrial gene expression pattern was very similar between stimulated and natural cycles. This delay of endometrial receptivity in stimulated cycles explains the difference of endometrial gene expression profiles observed during the WOI when comparing the profiles at hCG + 7 of COS versus LH + 7 of a previous natural cycle [23–25, 40]. Horcajadas et al. [24] reported that genes up-regulated during the formation of the receptive endometrium in the natural cycle tended to be down-regulated during stimulated cycles [24]. This finding was confirmed by the same group in 2008 [40]. They showed that a high number of genes was found to be differently expressed between natural and COS (GnRH agonists or antagonists). However, another study disagreed with these findings and reported no significant changes in gene expression profile when comparing LH + 8 and hCG + 9 [27].

In their review on ER in 2012, Haouzi et al. [7] emphasized the existence of a common transcriptomic profile during the endometrial shifts from the pre-receptive to the receptive stage between natural and stimulated cycles and a specific endometrial transcriptomic signature associated with COS protocols. In natural as well as in stimulated cycles, the majority of the genes modulated during the WOI were up-regulated (93% in natural compared with 78% in stimulated cycles). Among the up-regulated genes in the stimulated cycles during the WOI, only 46% were in common with those of the natural cycles. These data suggest that either the duration or FSH dose in gonadotrophin treatment under COS cycles leads to the transcriptional activation of other genes which are not involved in physiological endometrium receptivity [7, 23]. On the other hand, several studies agree that COS protocols affect ER despite some contradictions in terms of the impact level of the protocols on the endometrium. Indeed, some groups have shown that both long GnRH-agonist and antagonist protocols only slightly affect ER compared with natural cycles [23, 27, 29], while others have provided evidence of a strong impact of these protocols on ER [23, 47, 48]. In light of this situation, two endometrium gene profiles were observed and associated either with a moderately altered receptivity for the majority of the patients (86%) or with a strongly altered receptivity in a few cases (14%), when the pre-receptive and receptive phases in stimulated (hCG + 2 and hCG + 5) and natural cycles (LH + 2 and LH + 7) for the same patient were compared [23]. In these few cases the transition from the oocyte retrieval day (hCG + 2) to the embryo transfer day (hCG + 5) introduced only minor differences in gene expression profiles. Only one gene, Tetraspanin-3, was significantly modulated between the pre-receptive to receptive samples.

The samples studied were retrieved in the same fertile women, which is important to minimize inter-patient variability and suggest that true differences are not masked. These results are consistent with those obtained by another study that demonstrated that COS protocols only slightly affect ER compared with natural cycles [27, 29]. Mirkin et al. observed only a small variation in gene expression between natural and stimulated cycles, with 18 genes showing changes in expression [27]. However, other studies have provided evidence of a strong impact of COS regimens on ER [47]. Indeed, these studies demonstrated that COS can dysregulate the expression of many genes involved in cell adhesion, T-cell receptor signaling, and regulation of signal transduction [47]. Moreover, another study demonstrated considerable dysregulation of genes coding for factors known to be important regulators of ER, such as glycodelin and LIF [48]. Differences in endometrial chemokines and growth factors under stimulated cycles in comparison with natural cycles were observed [44].

On the other hand, antagonists and agonists treatments have been compared in fertile women [27, 29, 30]. Haouzi et al. compared the effect of both GnRH antagonist and agonist long protocols on the ER by analyzing he global gene expression profile shift from the pre-receptive to the receptive stages of stimulated cycles versus natural cycles for the same fertile patients [30]. They demonstrated that ER under the GnRH antagonist protocol was more closely aligned with the natural cycle receptivity than under the GnRH agonist protocol (43% vs. 15%). These results support previous findings suggesting that GnRH antagonist protocols mimic the natural endometrial receptivity more closely than GnRH agonist long protocol [27, 29]. They also concluded that significant changes were found when comparing cycles using GnRH agonist versus antagonist (13 significantly different genes) [27].

The divergence between the reports analyzing the effects of stimulation protocols on ER may be explained by several reasons listed in Table 1, especially differences in the day of the endometrial biopsies, different COS protocols and inadequate numbers of endometrial samples studied.

## Transcriptomics of endometrial receptivity of infertile women

In the last few years some groups have been interested in learning whether women with unexplained infertility and/or repeated IVF failure have a different genetic profile during the WOI in natural cycles or stimulated cycles using microarray technologies [25, 31, 34, 36, 42, 49, 50].

### Natural cycles

Few studies have compared the gene-expression profile of human ER at the time of embryo implantation of infertile versus fertile patients in natural cycle. All these studies demonstrated a different endometrial gene expression pattern between the two groups. Moreover, a clear distinction is present between the two groups in the up-regulated genes, as well as with those that were down-regulated [31, 34, 42].

As shown in Table 2, three specific transcriptomic signatures that can predict recurrent implantation failure (RIF) were identified. Altmäe et al. reported a specific transcriptomic signature of 260 genes including 145 up- and 115 down-regulated genes in the endometria of infertile versus fertile women during the WOI under natural cycles [31]. In contrast, Koler’sgroup determined that more than 300 genes exhibited a modified expression profile in RIF, 288 of these genes were down-regulated and 25 genes were up-regulated [42]. This is consistent with a recent study of RIF that also reported a molecular signature containing 303 genes [34]. All these studies detected a substantial number of dysregulated genes and pathways in the endometrium of infertile women [31, 34, 42]. Indeed, Altmäe et al. detected a substantial number of dysregulated genes in the endometrium of infertile women, the latter being involved primarily in cellular localization, transport, transporter activity.

Also, they showed dysregulation of pathways involved in leukocyte extravasation signalling, lipid metabolism and detoxification in the endometrium of infertile women [31]. Furthermore, the classification of the down-regulated genes to biological pathways by Koot et al. revealed dysregulation of specific interest pathways for implantation, the cell cycle, the cellular adhesion and the Wnt pathways [42]. These findings are also in accordance with Koot et al. who concluded that RIF is primarily associated with reduced cellular proliferation [34]. However until now, none of these specific transcriptomic signatures predicting recurrent implantation have been commercialized.

### Stimulated cycles

There are few recent studies comparing the gene expression of the endometrium in stimulated cycles of infertile versus fertile women at the time of embryo implantation [25, 36, 50]. As demonstrated in fertile women with stimulated cycles, the majority of these studies showed that COS modulates the gene expression profiles of human endometrium, affect ER and causes a displacement of the WOI of infertile women.

Liu et al. [25] compared the effect of COS on the gene expression patterns of endometrium in infertile patients with normal menstrual cycles in stimulated cycles (hCG + 7) versus natural cycles (LH + 7) [25]. They demonstrated that COS caused aberrant expression of a number of genes in human endometrium affecting the regulation of several biological pathways. Some of these pathways have been shown to play important roles in the implantation process. They observed changes in the expression of the genes involved in adherent junctions, complement and coagulation cascades, cytokine-cytokine interaction, eicosanoid synthesis, Wnt signaling, MAPK signaling pathway, and nicotinamide metabolism. This is consistent with a recent study of Altmäe et al. [50] which analyzed gene expression profiles in infertile women undergoing two different endometrial preparation protocols; stimulated and natural cycle with FET versus fertile women in natural cycle. They demonstrated that natural cycles are associated with a better ER than stimulated cycles. Stimulated cycles seemed to have a stronger negative effect on gene expression implicated in crucial pathways of ER. Moreover, using the ERA prediction tool, Ruiz-Alonzo et al. confirmed that the displacement of the WOI is more frequent in RIF patients both in COS or natural cycles than in fertile women [36]. These findings are also in accordance with Haouzi’s study [33], using the Win-test, they found that endometrium was non-receptive (29 and 43% in oocyte donation (OD) recipient and RIF OD recipient patients, respectively) or partially receptive (71 and 43% in OD recipient and RIF OD recipient patients, respectively), at Pg + 5/ + 6, in the majority of hormone replacement therapy treated patients.

Overall, in natural or COS cycles, transcriptomic modification of the endometrium occurs in RIF patients during the WOI which could be due to a displacement of the WOI and/or pathologic alteration. Furthermore, there is a clearly increased proportion of WOI displacement in RIF patients compared with fertile patients.

## Conclusions

In 15 years of transcriptomic analysis on endometrial receptivity, we have learnt that there is a specific transcriptomic profile during the WOI which is affected or delayed by COS in fertile and infertile patients. COS can slightly or strongly affect ER compared with natural cycles. However, ER under the GnRH antagonist protocol corresponds more closely with natural cycle receptivity than under the GnRH agonist protocol. The endometrial gene expression pattern at the time of embryo implantation in infertile women differs from that of fertile women; indeed the proportion of WOI displacement is higher in infertile compared with fertile patients. These data highlight the necessity to detect the WOI of IVF patients and eventually adjust the embryo transfer day according to the shift in the WOI, particularly in patients with multiple implantation failures. In this case, a clinician could chose to adapt the IVF stimulation protocol or counsel patients to either a fresh or frozen embryo transfer, depending on the impact level of the COS protocols on the endometrium.

The large range of results of genes expression profiles associated with the WOI lead us to conclude that we do not yet have a complete understanding of all the interactions between the embryo and the endometrium at the time of implantation**.** Regardless of all these transcriptomic studies, only two signatures became clinically validated tests. The ERA Test [20] and the Win-Test [21], were designed to evaluate ER and to personalize the FET, however, these two genomic diagnostic tools are not comparable from an analytical point of view. ERA is a customized array based on the expression of 238 genes coupled to a computational predictor capable of diagnosing a functionally receptive endometrium, whereas the Win-Test is based on the quantitative expression of 11 predictive genes of ER coupled with an algorithm allowing for the identification of the receptive state. Available data on fresh (stimulated cycles) and frozen cycles (natural/hormonal replacement cycles) showing no differences in pregnacy or implantation rates.A recent studies comparing the fertility success rate in fresh versus frozen embryo transfer, reported that no significant difference in clinical pregnancy rates, implantation rates, or ongoing pregnancy rate between the fresh and the frozen embryo transfer groups [51–53]. These findings suggest that new biomarkers for exploration of endometrial receptiveness are highly conserved that’s why they are proposed in natural and stimulated cycles. Expression of this genes is not affect by COS is just delayed hence the delay of WOI. In their review on ER in 2012, Haouzi et al. emphasized the existence of a common transcriptomic profile during the endometrial shifts from the pre-receptive to the receptive stage between natural and stimulated cycles. Highly conserved endometrial receptivity biomarkers have been identified from this common transcriptomic signature between natural and stimulated cycles.

Although, these two studies have observed modifications in gene expression profile associated to the transition of the human endometrium from a pre-receptive to a receptive in fertile women without considering the fact that these women might have a variation in the WOI. Moreover, a recent study demonstrated that pregnancy rate in patients who underwent FET is the same following an adjustment in timing of FET according to the ERA test [54]. Other studies questioned the consistency of the diagnosis of the WOI using the ERA test after variability observed from month to month following various biopsies in one patient [55, 56].

Successful implantation is a complex process requiring a receptive endometrium, a viable embryo and synchronized dialogue between maternal and embryonic tissues. Despite 15 years of transcriptomic analysis on endometrial receptivity more studies are needed to optimize the selection of biomarkers of ER and maternal-fetal dialogue that influence pregnancy outcome. Without forgetting that there are many other factors that can influence the pregnancy rate such as the physician or the embryologist performing the transfer, difficulties in inserting the transfer catheter, endometrial thickness and pattern, and subendometrial contractions. Those factors were not controlled in those studies [57].

## Data Availability

“Not applicable”.
